# Photonics bridges between turbulence and spin glass phenomena in the 2021 Nobel Prize in Physics

**DOI:** 10.1038/s41377-022-00793-w

**Published:** 2022-04-21

**Authors:** A. S. L. Gomes, C. B. de Araújo, A. M. S. Macêdo, I. R. R. González, L. de S. Menezes, P. I. R. Pincheira, R. Kashyap, G. L. Vasconcelos, E. P. Raposo

**Affiliations:** 1 Departamento de Física, Universidade Federal de Pernambuco, Recife, PE 50670-901 Brazil; 2 Unidade Acadêmica de Belo Jardim, Universidade Federal Rural de Pernambuco, Belo Jardim, PE Brazil; 3 Chair in Hybrid Nanosystems, Nanoinstitut München, Fakultät für Physik, Ludwig-Maximilians-Universität München, 80539 München, Germany; 4 Departamento de Ciencias Físicas, Universidad de La Frontera, Temuco, Chile; 5 Fabulas Laboratory, Department of Engineering Physics, Department of Electrical Engineering, Polytechnique Montreal, Montreal, H3C 3A7 QC Canada; 6 Departamento de Física, Universidade Federal do Paraná, Curitiba, PR 81531-990 Brazil

**Keywords:** Optics and photonics, Physics

## Abstract

A photonic connection between turbulence and spin glasses has been recently established both theoretically and experimentally using a random fiber laser as a photonic platform. Besides unveiling this interplay, it links the works of two 2021 Nobel laureates in Physics.

The 2021 Nobel Prize in Physics was awarded to Klaus Hasselmann, Syukuro Manabe, and Giorgio Parisi for their “groundbreaking contributions to our understanding of complex physical systems.” One of the striking issues featured by the Nobel Committee for Physics in the “Scientific Background” document^1^ was the recent unveiling of the intricate interplay between turbulence and spin glasses, two of the most elusive physical phenomena that lie at the very heart of the seminal works by Hasselmann and Parisi, respectively.

In a series of articles^2–4^ using a random fiber laser (RFL) as a photonic experimental platform, our group first characterized^2^ the spin glass phase with replica symmetry breaking (RSB) above the RFL threshold, and then discovered^3^ in the same set of measurements a simultaneous photonic turbulent-like regime of emitted intensities, successfully described by a hierarchical stochastic model including Kolmogorov’s tenets for turbulence, viz. energy cascade and intermittency. We finally built in^4^ the connection between these complex photonic behaviors through a modified Pearson coefficient comprising both turbulent and spin glass phases.

RFLs are the quasi one-dimensional realization^5^ of random lasers (RLs), a class of disordered photonic systems proposed early in 1968 but unambiguously demonstrated only in 1994, as reviewed in^6^. RLs differ from conventional lasers as their operation does not require a conventional optical cavity. Rather, the optical feedback for laser action arises from multiple light scattering by elements (atoms, ions, nano- or micro-particles) randomly located within the lasing medium. In^2–4^ we have employed a specially designed erbium-doped fiber with a huge number of inscribed random fiber Bragg gratings, resulting in an open disordered complex system with suitable gain and scattering to provide the optical feedback for RL action.

The spin glass phase was discovered in 1972 in disordered magnetic materials^7^. Since then, an impressive sequence of noteworthy advances took place^8^, which ultimately led to the introduction by Parisi of the seminal concept of RSB^9,10^. To cut a long history short, Parisi’s theory relies on the fact that the interplay between disorder and magnetic frustration below some critical (freezing) temperature yields a free energy landscape with a great number of local valleys and maxima that can trap distinct replicas of the spin glass system for relatively long times, leading to different measures of observables and irreversibility effects. This feature sharply contrasts with the two degenerate minima in the free energy of a replica-symmetric disorder-free ferromagnet. On the photonic side, the RSB glassy behavior of light was theoretically proposed in 2006^11^ and demonstrated experimentally in 2015^12^ in a RL with two-dimensional geometry. Shortly after, it was also reported in a RFL^2^.

Turbulence is also a fascinating multidisciplinary subject^13^, which has lately found a promising platform in optics and optical fiber-based systems^14,15^. As mentioned, our group demonstrated^3^ turbulent-like behavior in the first RFL in which an RSB glassy phase was found^2^. We analyzed^4^ turbulence and RSB glassy effects jointly through a modified Pearson correlation coefficient $Q_{\tau ,\alpha \beta }$ simultaneously sensitive to both photonic glassy and turbulent behaviors. By labeling as *α* and *β* two RFL spectra emitted under identical conditions (system replicas), the variable $x_{\tau ,\alpha }\left( k \right) = I_{\alpha + \tau }(k) - cI_\alpha (k)$ denotes the emitted intensity in mode *k* if *c* = 0 for *τ* = 0, and the intensity fluctuation of spectra separated by *τ* time units if *c* = 1 for *τ* > 0. We define $Q_{\tau ,\alpha \beta }$ as the Pearson covariance^4^ of pairs of variables $x_{\tau ,\alpha }\left( k \right)$ and $x_{\tau ,\beta }\left( k \right),\alpha \,\ne \,\beta$, normalized by the standard deviations. The distributions $P(Q_{\tau ,})$ and $P(x_\tau )$ characterize the emergence of the RSB spin glass and turbulent-like intermittency phenomena (see^4^ for details). As illustrated in Fig. 1, below threshold the erbium-doped RFL displays a prelasing non-turbulent regime with replica symmetry, while above threshold a photonic RSB spin glass phase sets in concomitantly with turbulent-like behavior.

**Fig. 1:**
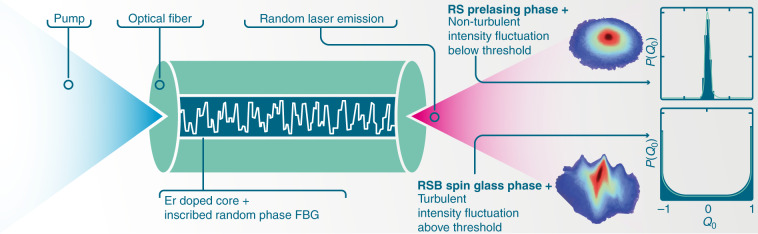
Schematic erbium-based RFL with random fiber Bragg gratings. Below threshold, a single central maximum in the distribution $P(Q_\tau )$ for *τ* = 0 of the Parisi replica overlap parameter indicates a replica-symmetric (RS) regime with non-turbulent behavior identified by a Gaussian $P(x_\tau )$ for *τ* = 1. Above threshold, $P(Q_\tau )$ for *τ* = 0 displays two side maxima demonstrating the presence of a RSB spin glass phase simultaneous with turbulent-like behavior in which $P(x_\tau )$ for *τ* = 1 is a heavy-tailed distribution of asymptotic stretched-exponential form. Intermittency effects fade away either below threshold or for $\tau \gg 1$, as rare events in $P(Q_\tau )$ at much shorter time scales become quite more likely for larger *τ* (Adapted from^4^)

In the words of Thomas Bohr and coauthors in 1998^16^, “The existence of a connection between fluid dynamic turbulence and spin glasses is fascinating, although it is still too early to decide whether it can pave the way to a deeper understanding of intermittency in fully developed turbulence.” It took 20 years for an experimental and theoretical verification of such connection in a photonic environment, namely an RFL system^4^. In this context, the relevance of the photonic bridge between two of the most intricate physical phenomena is stressed by the Nobel Committee for Physics on page 11 of Ref. ^1^: “Finally, the nature of the random laser system allows for the concomitant observation of replica symmetry breaking and connection between spin-glasses and turbulence [34], particularly nonlinear wave interactions, which link the early work of Hasselmann [40, 41] to that of Parisi and to the role of disorder and fluctuations in complex systems in general.” (Reference [34] above is our work^4^ and Refs. [40, 41] correspond, respectively, to^17,18^.)
